# Complete Genome Sequence of a Japanese Clinical Isolate of Haemophilus influenzae Type a Strain TAMBA230

**DOI:** 10.1128/MRA.01069-20

**Published:** 2020-11-25

**Authors:** Mayumi Kubota, Tsuyoshi Kenri, Yuko Sasaki, Keigo Shibayama, Kosuke Takayanagi, Tsuneaki Kenzaka

**Affiliations:** a Department of Bacteriology II, National Institute of Infectious Diseases, Tokyo, Japan; b Department of Clinical Laboratory, Hyogo Prefectural Tamba Medical Center, Hyogo, Japan; c Department of Internal Medicine, Hyogo Prefectural Tamba Medical Center, Hyogo, Japan; d Division of Community Medicine and Career Development, Kobe University Graduate School of Medicine, Hyogo, Japan; Georgia Institute of Technology

## Abstract

Haemophilus influenzae causes severe infections such as pneumonia and meningitis. Here, we report the complete genome of H. influenzae type a strain TAMBA230, which was isolated in 2019 from a patient exhibiting bacteremia. This represents the first case in Japan of an H. influenzae type a strain associated with invasive infection.

## Abstract (precis)

Haemophilus influenzae causes severe infections such as pneumonia and meningitis. Here, we report the complete genome of H. influenzae type a strain TAMBA230, which was isolated in 2019 from a patient exhibiting bacteremia. This represents the first case in Japan of a H. influenzae type a strain associated with invasive infection.

## ANNOUNCEMENT

Haemophilus influenzae, the first bacterium to undergo complete genome sequencing (1), is a Gram-negative coccobacillus of the family *Pasteurellaceae*. It can cause invasive infections (e.g., pneumonia and meningitis) and noninvasive infections (e.g., pharyngitis and otitis media) (2). This species encompasses six capsular polysaccharide-based serotypes (types a to f) and unencapsulated nontypeable (NTHi) strains. H. influenzae type b (Hib) strains were the major cause of invasive H. influenzae infections; however, infections by NTHi and strains of other serotypes have become more common since the introduction of Hib vaccines (3, 4).

Here, we report the complete genome sequence of a clinical isolate of H. influenzae type a (Hia) strain TAMBA230, the first instance of this strain causing a severe infection in Japan. Hia infections have been reported in North America, mainly among indigenous people (5, 6). The TAMBA230 strain was isolated from a 72-year-old Japanese male patient with pyogenic arthritis and bacteremia. The splenic function of the patient was normal. The bacterium was isolated from the patient’s blood by culture at 37°C overnight using chocolate II agar medium supplemented with factors V (NAD) and X (hemin) in the presence of 5% CO_2_. Colonies on the plate exhibited mucoid morphology. Capsular type was determined via analysis with anti-Hia rabbit serum and PCR-based assays (7, 8).

A single colony on the chocolate II agar medium was picked up, cultured to late log phase in *Haemophilus* test medium broth (Remel Oxoid, Lenexa, KS, USA) supplemented with NAD and hemin, and harvested by centrifugation. Genomic DNA was extracted by using a NucleoBond high-molecular-weight (HMW) DNA kit (Macherey-Nagel, Düren, Germany). Whole-genome sequencing was performed using the MiSeq (Illumina, San Diego, CA, USA) and GridION (Oxford Nanopore Technologies, Oxford, UK) systems. Libraries for Illumina sequencing (insert size, 400 to 1,000 bp) and Nanopore sequencing (insert size, >10 kb) were prepared using a Nextera DNA Flex library preparation kit (Illumina) and a Short Read Eliminator XS kit (Circulomics, Inc., Baltimore, MD, USA), respectively. Two MiSeq runs (2 × 156-bp paired-end reads) and a single GridION run produced 1,763,822 and 246,196 reads, respectively (average lengths, 154 and 5,889 bp, respectively; genome coverage, 150× and 801×, respectively). Raw read data were filtered for quality using fastp v0.20.0 and NanoFilt v2.7.1 tools for MiSeq and GridION data, respectively. The qualified data were assembled *de novo* using Unicycler v0.4.8 (9), followed by read alignment, resulting in a 1,809,645-bp circular genome with a G+C content of 38%. No plasmids or drug resistance genes were detected using PlasmidFinder v2.1 (10) and ResFinder v4.0 (11), respectively. The genome was annotated using the DDBJ Prokaryotic Annotation Pipeline (12). Software in these sequencing analyses was used with default settings unless otherwise specified.

The sequenced genome contains 1,694 coding sequences (CDSs), 19 rRNA genes (7 5S rRNA, 6 16S rRNA, and 6 23S rRNA genes), and 59 tRNA genes. The 16S rRNA sequences matched those of North American Hia strain M25588 (GenBank accession number CP031254) with 100% identity in a BLASTN analysis. The TAMBA230 strain genome encodes a multidrug efflux pump (*hmrM*) and 67 virulence factors. Although the TAMBA230 genome is approximately 20 kb smaller than that of the Hia strain NML-Hia-1 (GenBank accession number CP017811) (13), it is predicted to contain an additional 51 CDSs. Comparison by the Artemis Comparison Tool (ACT) revealed that there was an approximately 300-kb inversion in the TAMBA230 genome, compared to the NML-Hia-1 genome. Inspection of the inverted region sequence suggested that the inversion might have occurred by recombination between IS*3*-like sequences. Multilocus sequence typing (http://pubmlst.org/hinfluenzae) classified the TAMBA230 strain genome as sequence type 1511 (ST1511), whereas strain NML-Hia-1 is classified as ST23. This novel complete genome sequence represents a resource facilitating comparative genomic and epidemiological studies on H. influenzae.

This study was performed with the approval of the ethics committee of the National Institute of Infectious Diseases (approval number 884), and written informed consent was obtained from the patient.

### Data availability.

This whole-genome project has been deposited in DDBJ/ENA/GenBank under accession number AP022846, BioProject number PRJDB9304, and BioSample number SAMD00204789. The raw data have been deposited in the DDBJ Sequence Read Archive (DRA) under accession number DRA010758. The accession number for the Illumina sequence data is DRR243751, and that for the Nanopore sequence data is DRR243752.
